# Supplementary material for: Machine learning enables design automation of microfluidic flow-focusing droplet generation
Source: Nat Commun. 2021 Jan 4;12:25. doi: 10.1038/s41467-020-20284-z (PMC7782806; doi:10.1038/s41467-020-20284-z)
Supplement: Supplementary file 1 — Supplementary Information [file 41467_2020_20284_MOESM1_ESM.pdf]

# Machine Learning Enables Design Automation of Microfluidic Flow-Focusing Droplet Generation

Ali Lashkaripour, Christopher Rodriguez[†], Noushin Mehdipour[†], Rizki Mardian[†], David McIntyre[†], Luis Ortiz, Joshua Campbell, and Douglas Densmore

October 16, 2020

† These authors contributed equally.

## Supplementary Material

# 1 Supplementary Figures:

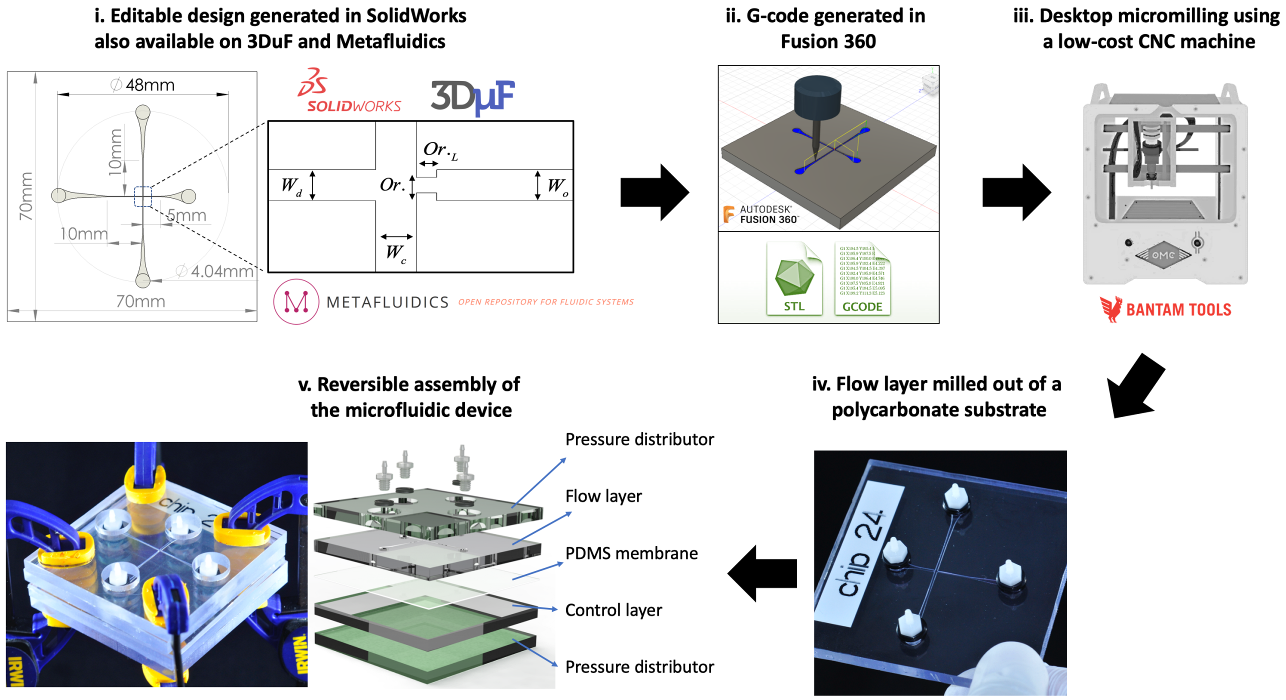


Supplementary Figure 1: Rapid prototyping is used to fabricate microfluidic devices. **i.** A standard size microfluidic device (7cm by 7cm) was used to create all the flow-focusing geometries. The designs were created in SolidWorks and were parameterized with six geometric parameters. Therefore, using the SolidWorks design file just by changing the value of the parameters a new design can be created without the need to edit or redraw any other part of the design. The parameterized design can be opened up in an interactive microfluidic CAD tool called 3D$\mu$F and is available for download at Metafluidics. Therefore, designs suggested by the design automation tool can be opened in 3D$\mu$F to edit or save as SVG or JSON formats. **ii.** The designs can be loaded to Fusion 360 or directly on the software controlling the CNC mill (OtherPlan or BantamTools) to automatically create the tool path for micromilling, called GCODE. **iii.** Once the GCODE is created the low-cost CNC micromill (Bantam Tools) can be used to **iv.** fabricate the layers of the microfluidic device from a polycarbonate sheet (thickness of 5.56 mm). **v.** Once all the layers are milled out, a thin layer of PDMS and clamps are used to assemble and seal the device in a low-cost and reversible manner.

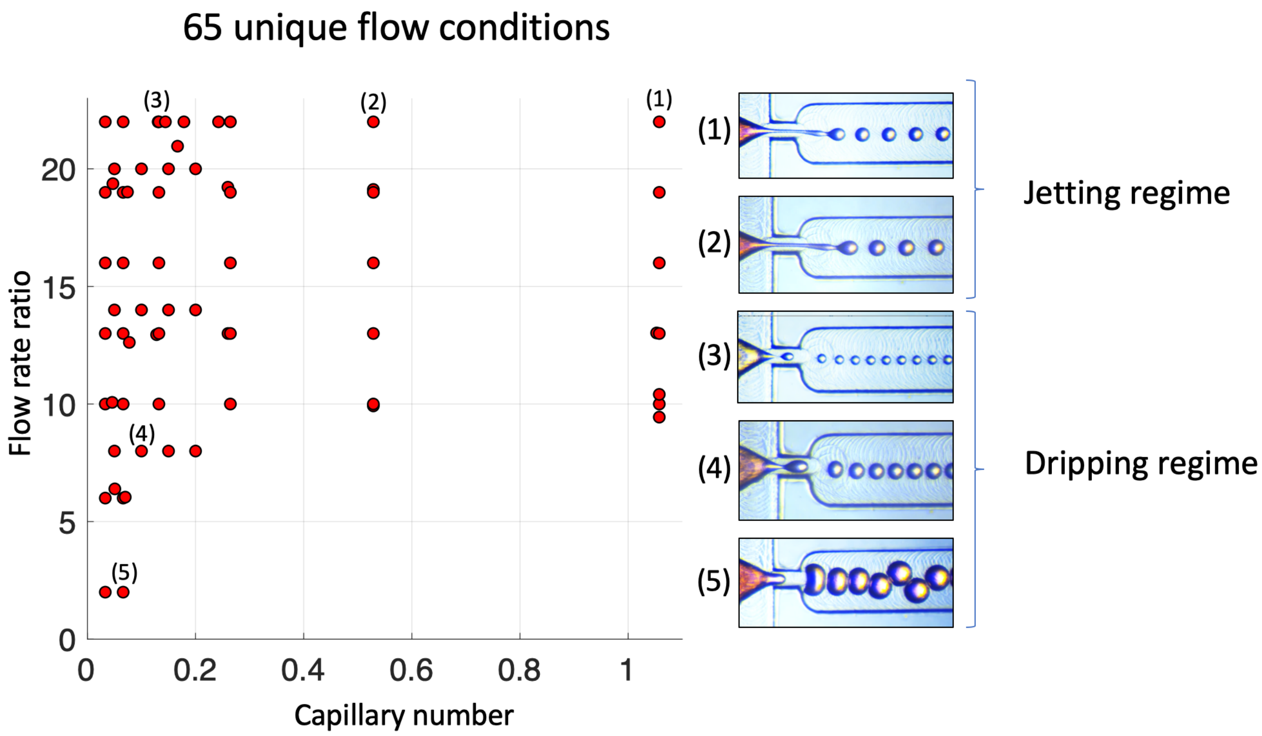


Supplementary Figure 2: Flow conditions used to generate the large-scale data-set. A total of 65 flow conditions were used to generate the large-scale data-set. The flow conditions were varied to achieve comparable number of data-points in both the dripping and jetting regimes. To generate a diverse data-set not all devices were tested at the same flow conditions.

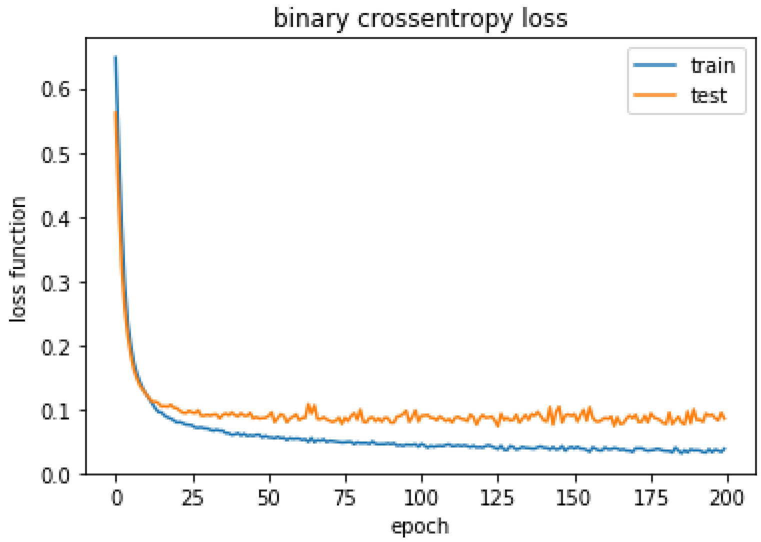


Supplementary Figure 3: Training performance of the classification neural network. Decrease in loss function for the optimized neural network classification model (predicting the droplet formation regime: dripping or jetting regimes) over the training epochs, with early stopping preventing the model from over-fitting to the train-set.

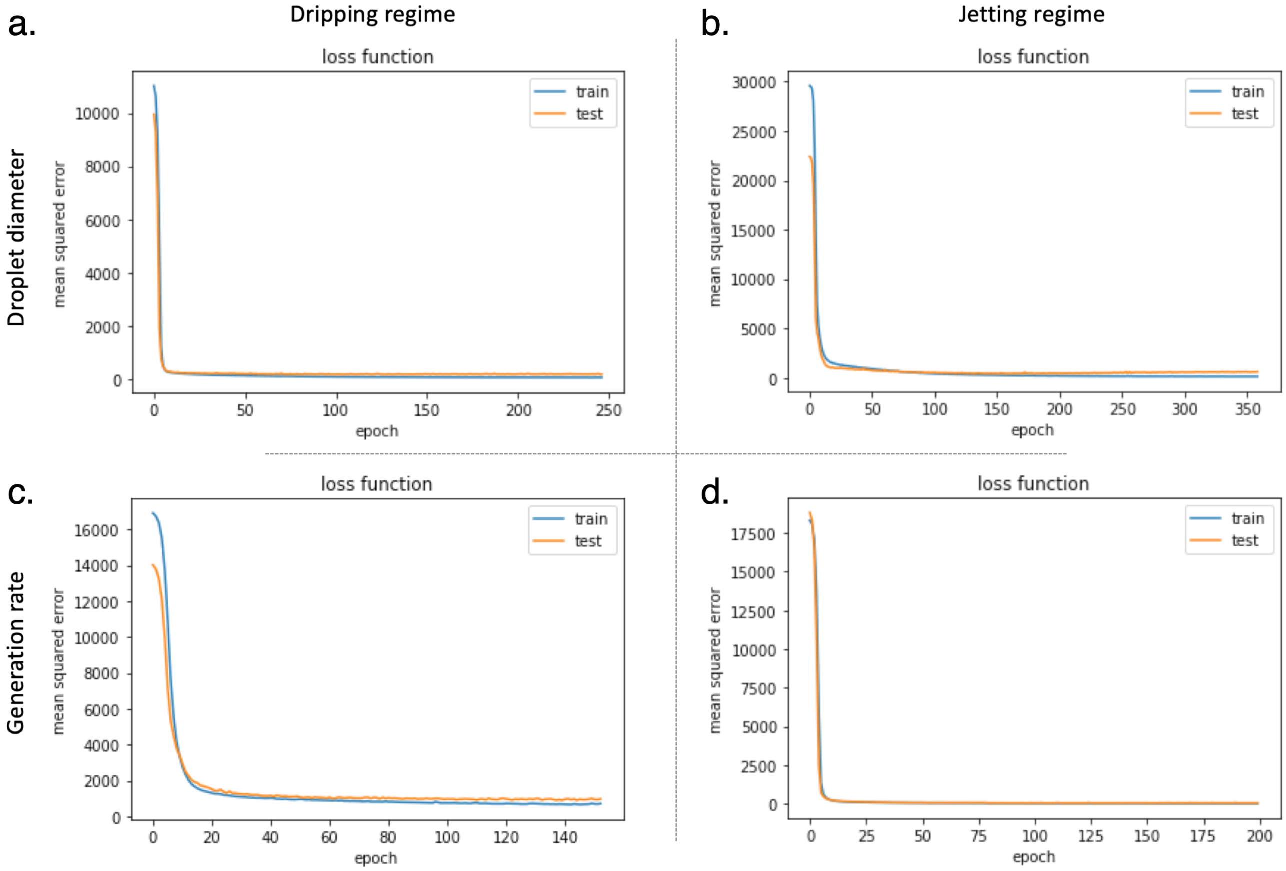


Supplementary Figure 4: Training performance of the regression neural networks. Decrease in loss function for the optimized neural network based regression models for predicting droplet diameter in **a.** dripping and **b.** jetting regimes and generation rate for both **c.** dripping and **d.** jetting regimes over the training epochs. By implementing early-stopping models were trained while avoiding over-fitting them to the train-set.

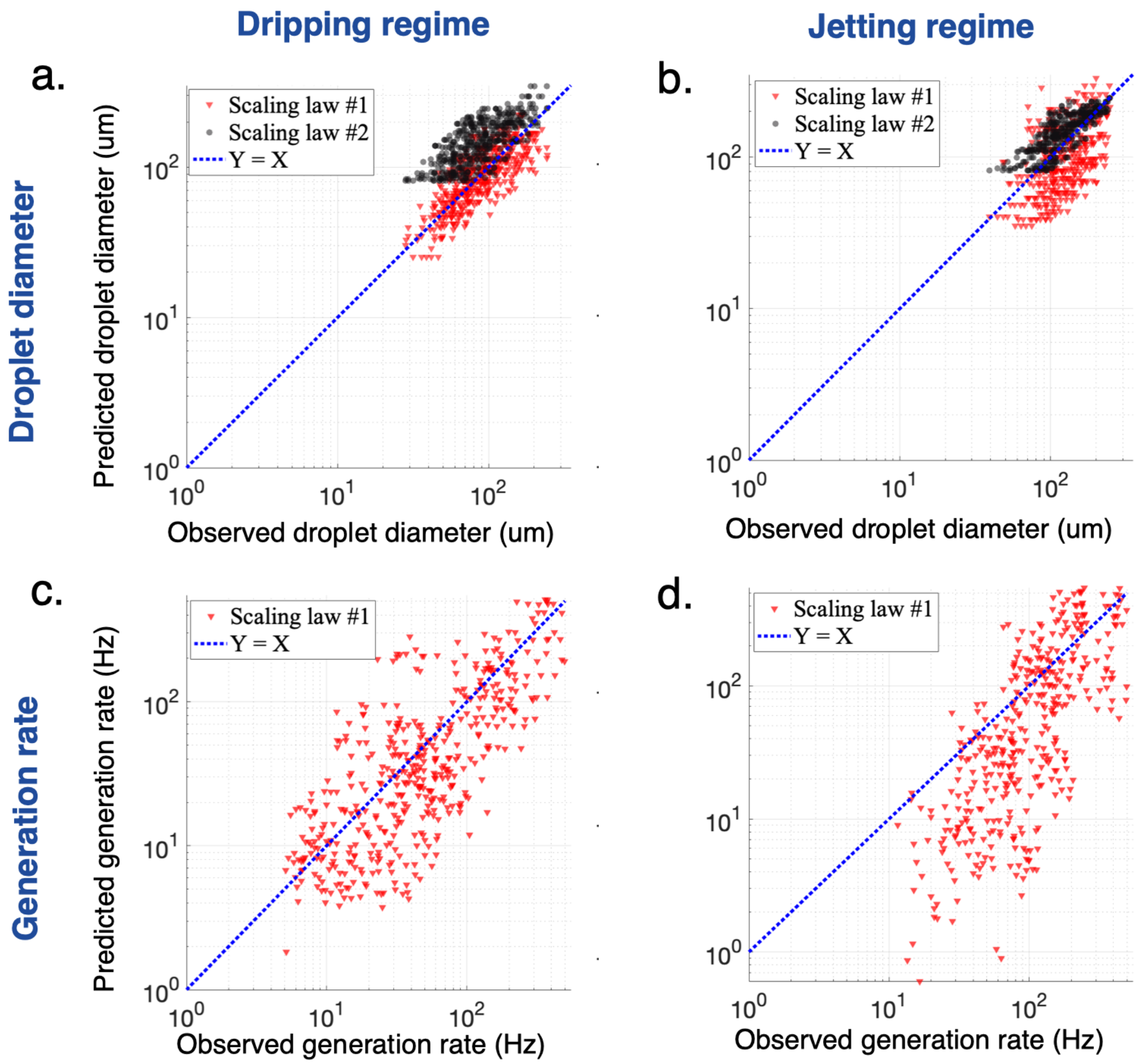


Supplementary Figure 5: Performance of scaling laws against the generated data-set. Recreation of Fig. 3 of the main manuscript for assessing the performance of the scaling laws against our data-set for droplet diameter in **a.** dripping and **b.** jetting regimes and for generation rate in **c.** dripping and **d.** jetting regimes in Log-Log scale.

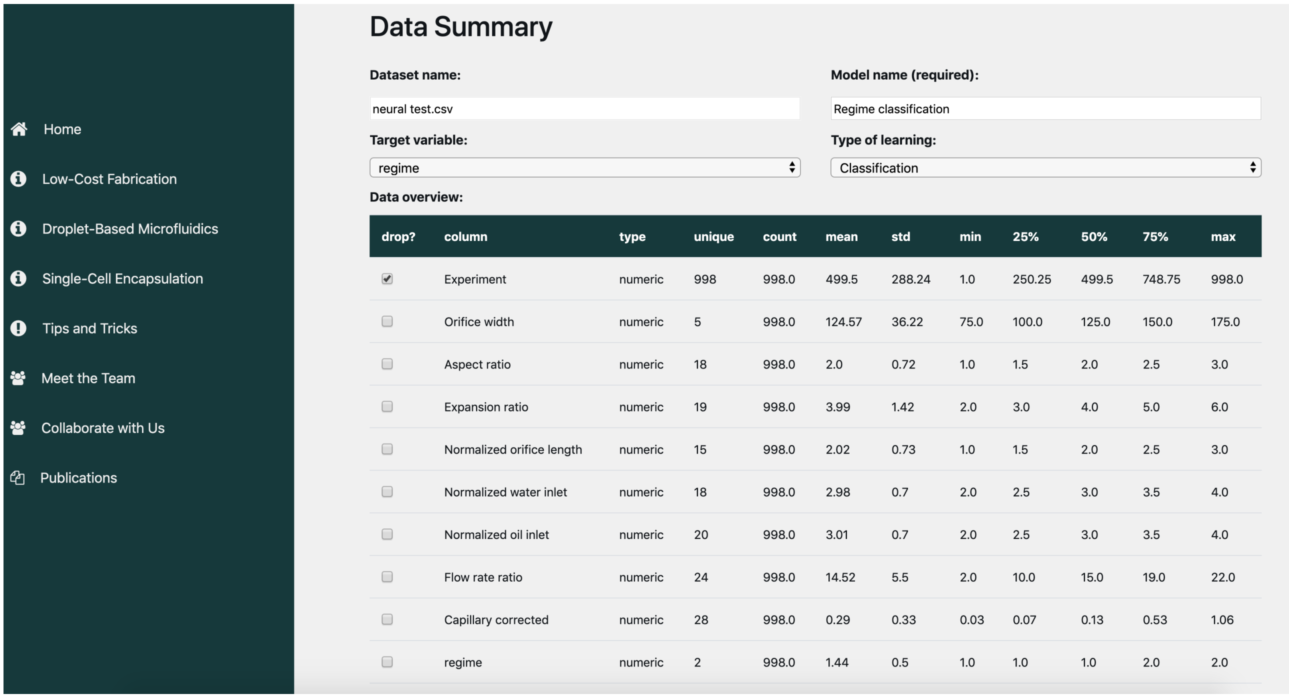


Supplementary Figure 6: The user-interface of DAFD Neural Optimizer. This online tool allows for an automated and easy-to-use data-to-model approach in machine learning. Researchers with a limited knowledge of machine learning can easily upload a data-set to DAFD website, and specify the type of learning (classification or regression), the inputs, and the output of interest, in order to build optimized neural networks that can predict the output based on the inputs.

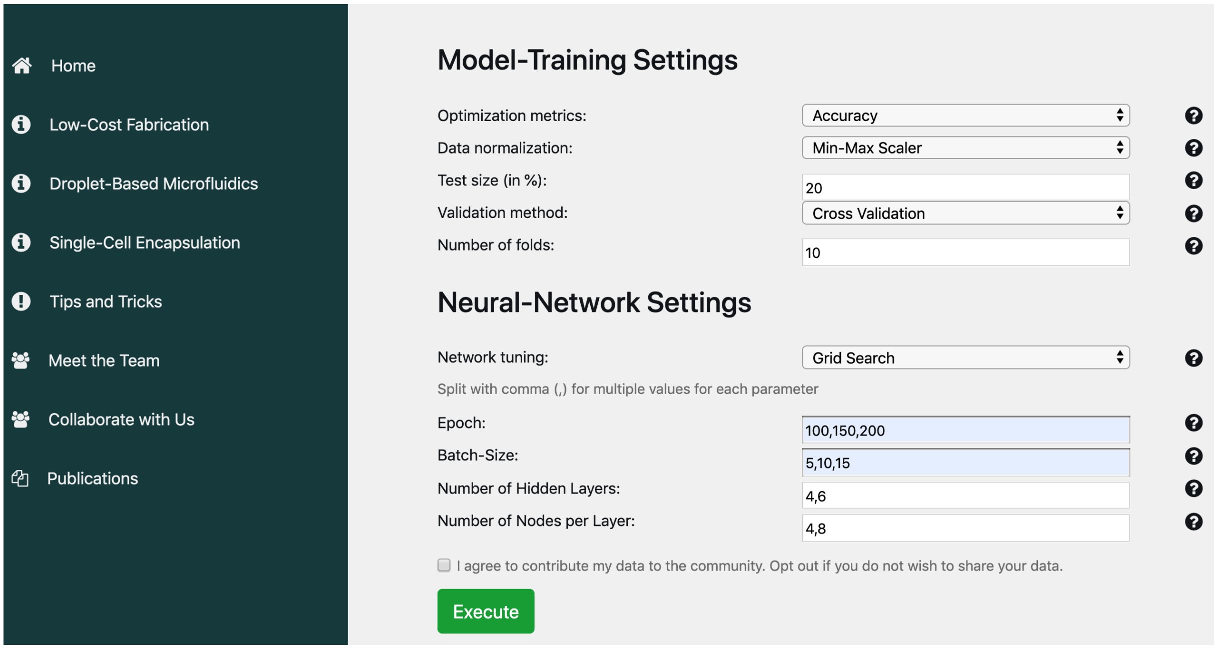


Supplementary Figure 7: Neural Optimizer allows for the search-space to be specified. After uploading a data-set to DAFD, Neural Optimizer user-interface allows researchers to choose the optimization metric, data-normalization method, test-set size, validation method, and the number of folds. Additionally, users can specify the the search space for hyper-parameters (i.e., number of epochs, batch-size, number of hidden layers, and number of nodes per layer) where Neural Optimizer will search (can be grid or random search) to find the optimal neural network hyper-perimeters (both structure and weights).

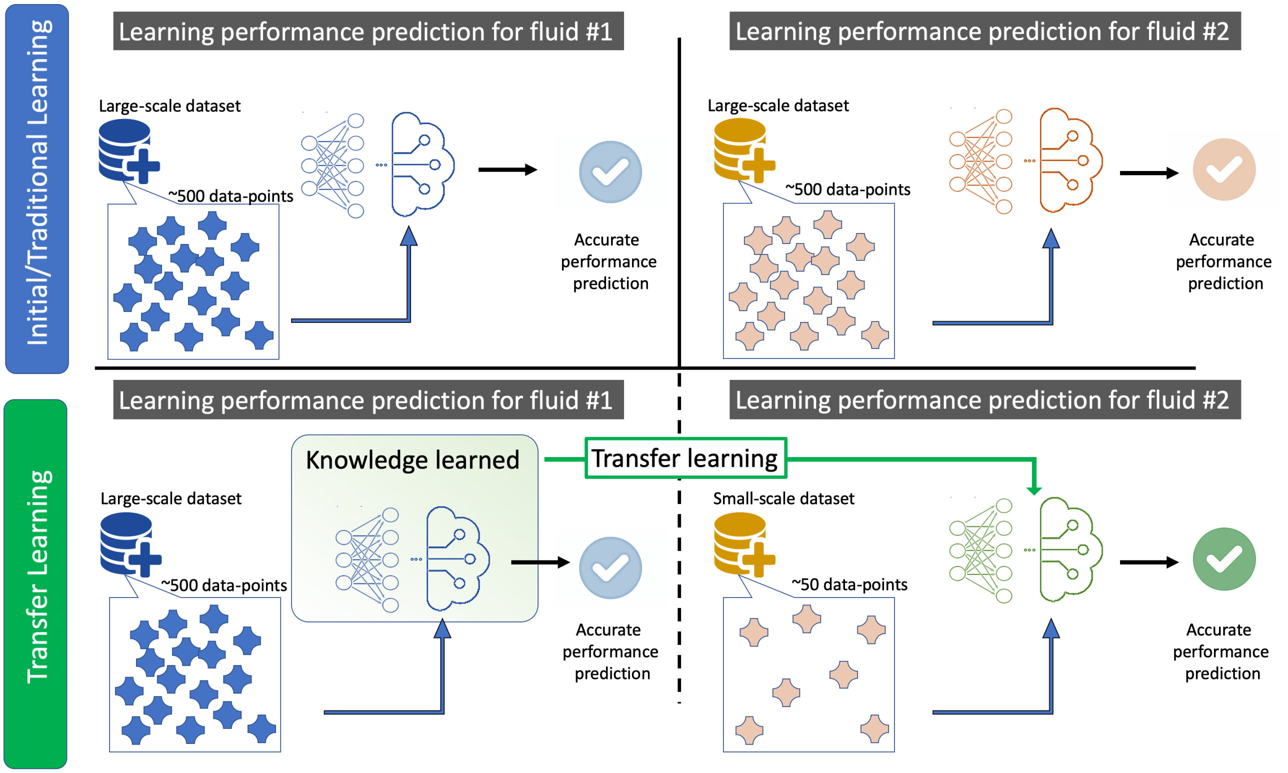


Supplementary Figure 8: Visualization of transfer learning concept. Transfer learning reduces the number of data-points required to train accurate predictive models on new fluid combinations.

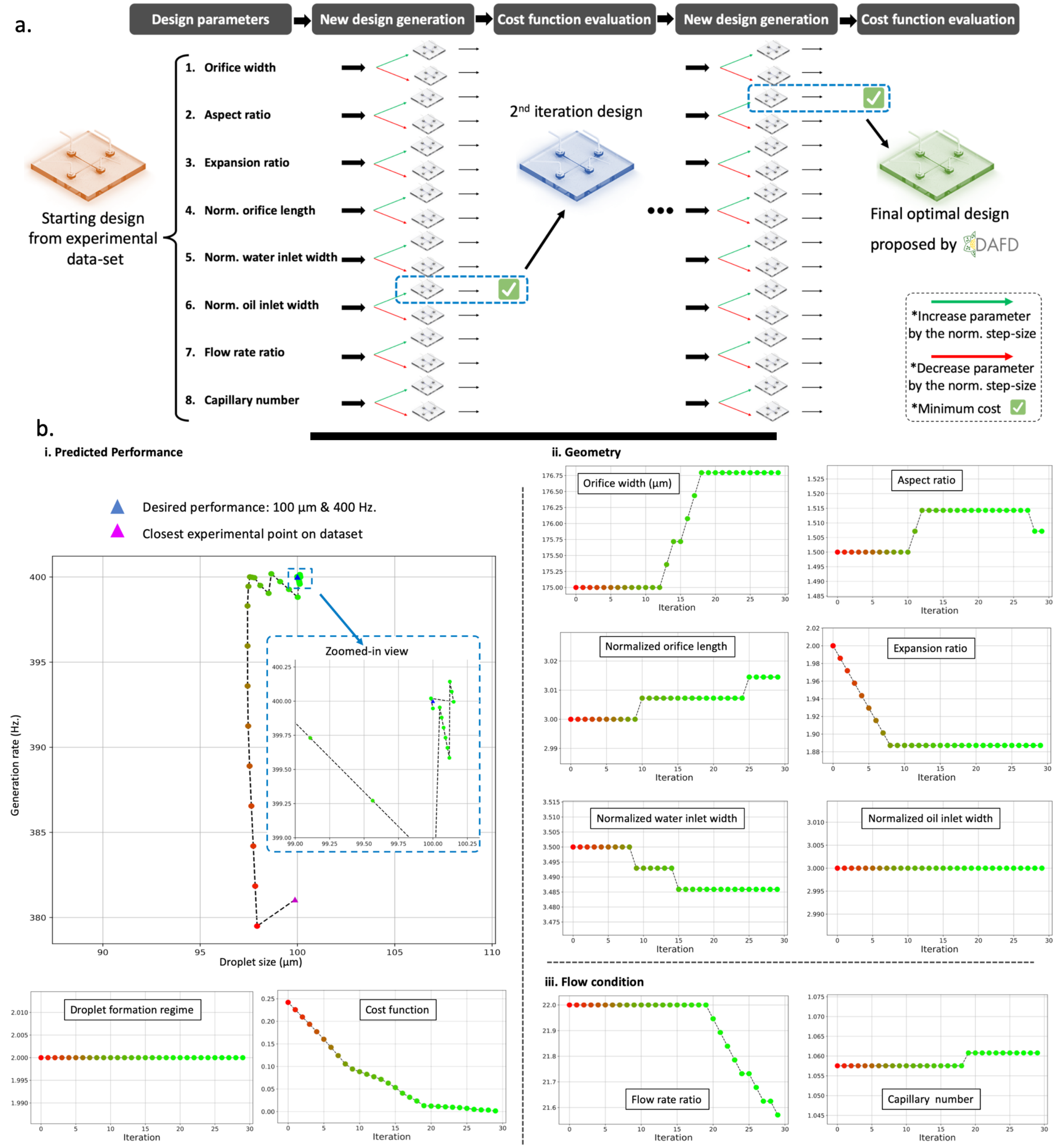


Supplementary Figure 9: Example of the design automation search procedure. **a.** The design automation process starts with finding a design on the date-set that most closely matches the desired performance. 8 design parameters are then altered to create 16 new designs. A cost function is evaluated to find the optimal design in the $1^{st}$ iteration. Then, the algorithm takes the previous optimal design as the starting design for the next iteration. This process is repeated until the cost function reaches zero or a minimum, and the optimal design that would result in the desired performance is found. **b.** Shows an example of how the design automation algorithm performs to reduce the cost function.

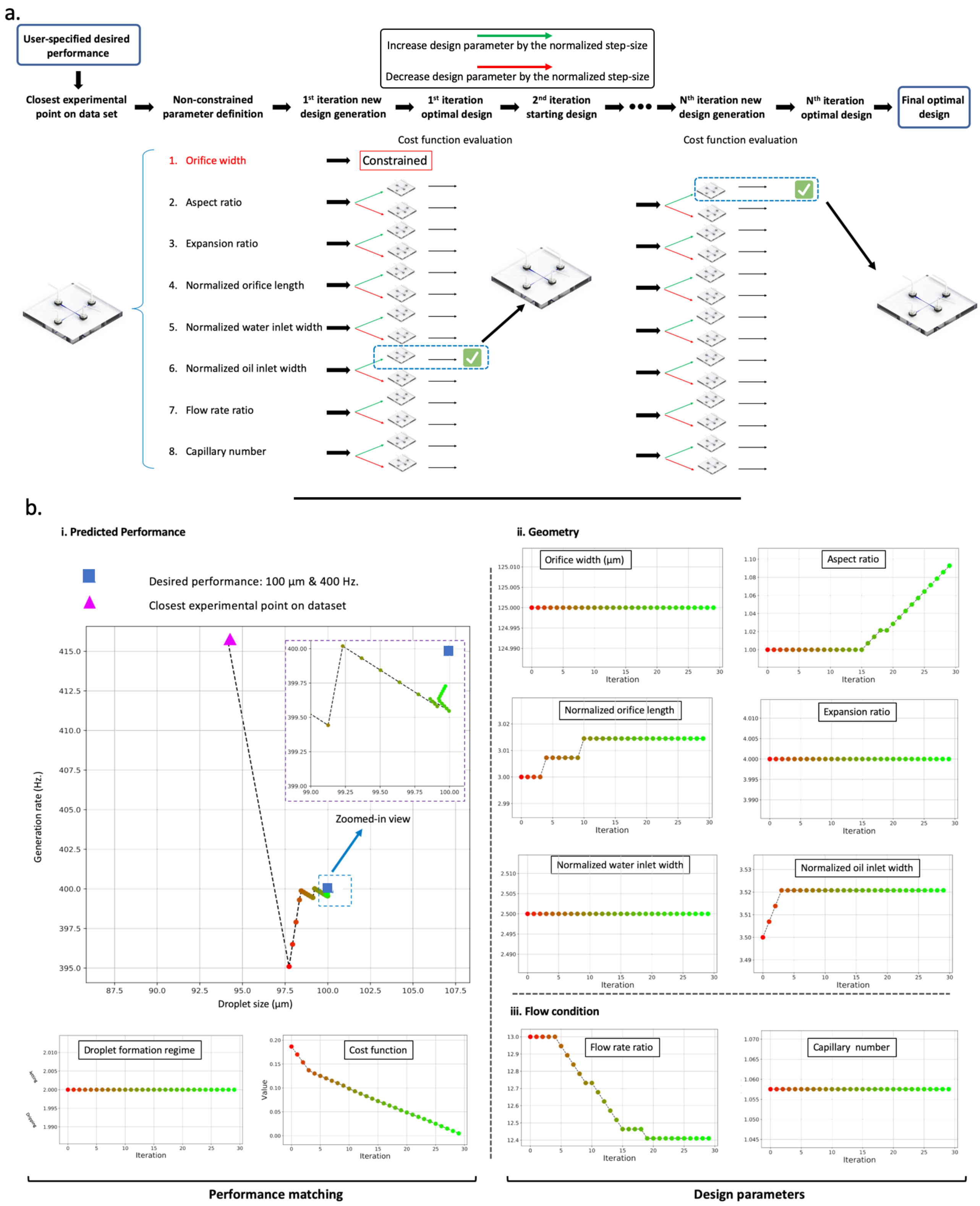


Supplementary Figure 10: Example of the design automation search procedure. This example of design automation workflow includes a user-specified constraint (Orifice width is constrained to 125 $\mu$m).

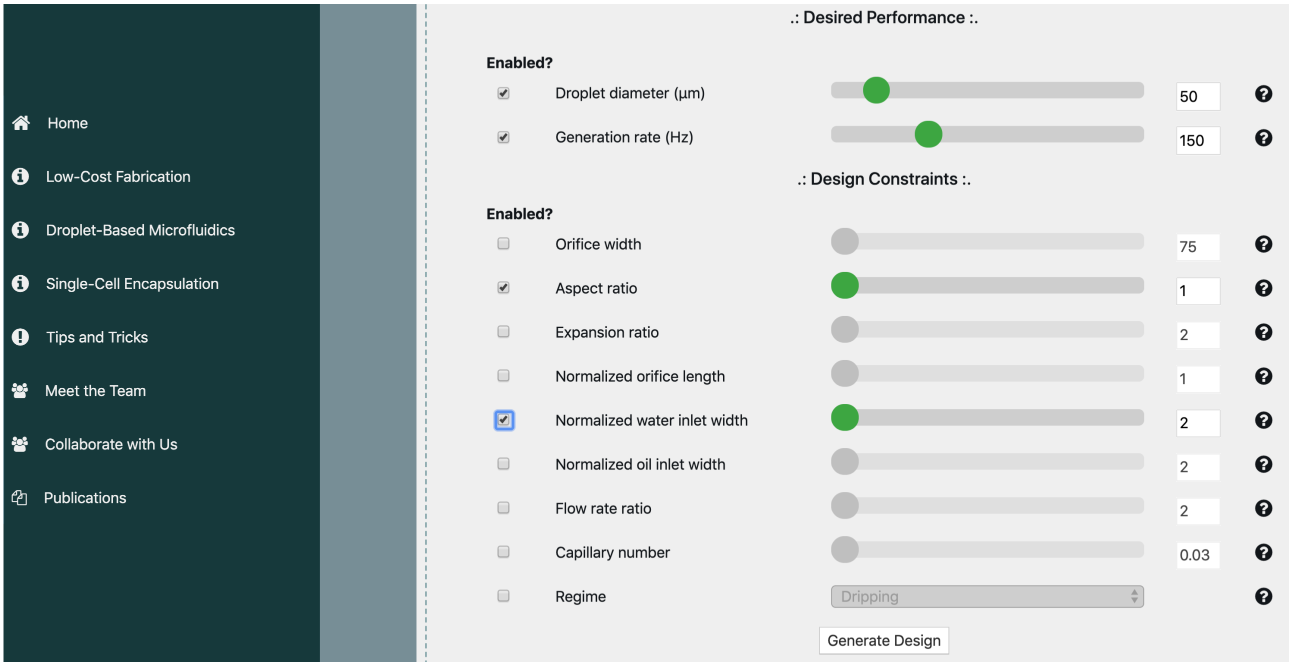


Supplementary Figure 11: A web-based tool for performance and constraints specification.

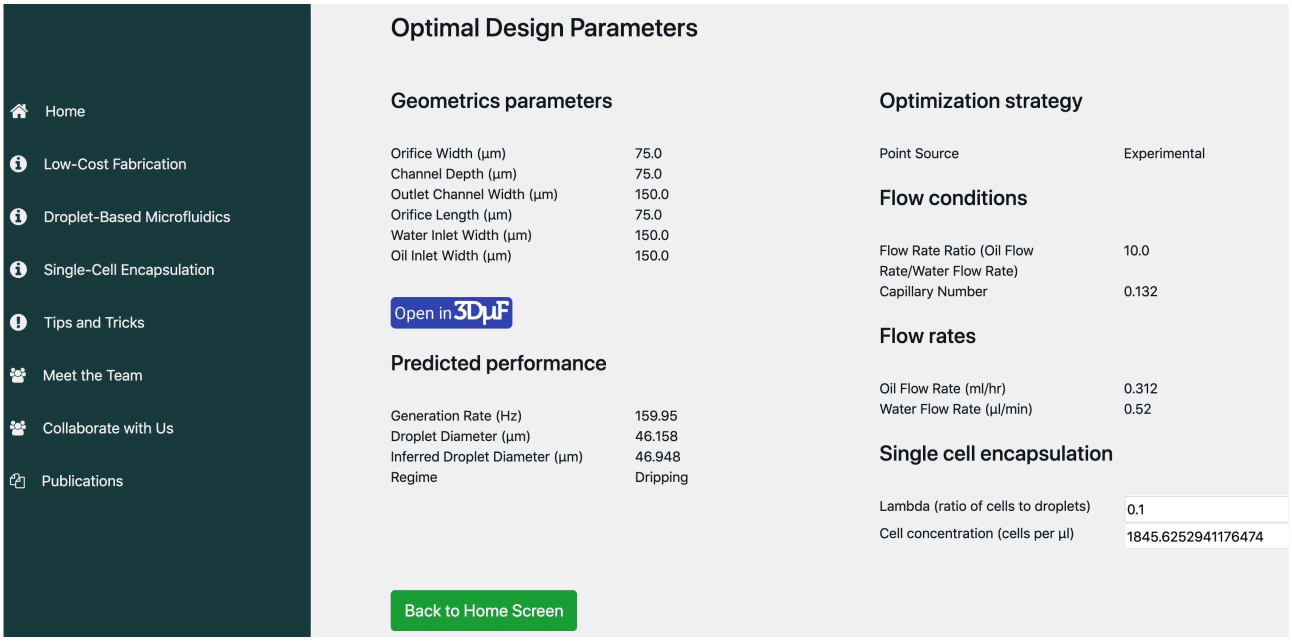


Supplementary Figure 12: An example of the the design automation tool's output. The design parameters to achieve the desired performance are provided by the tool. The generated design can be easily opened in 3D$\mu$F to edit the design, save it as svg files, and load them to the software controlling CNC micromill.

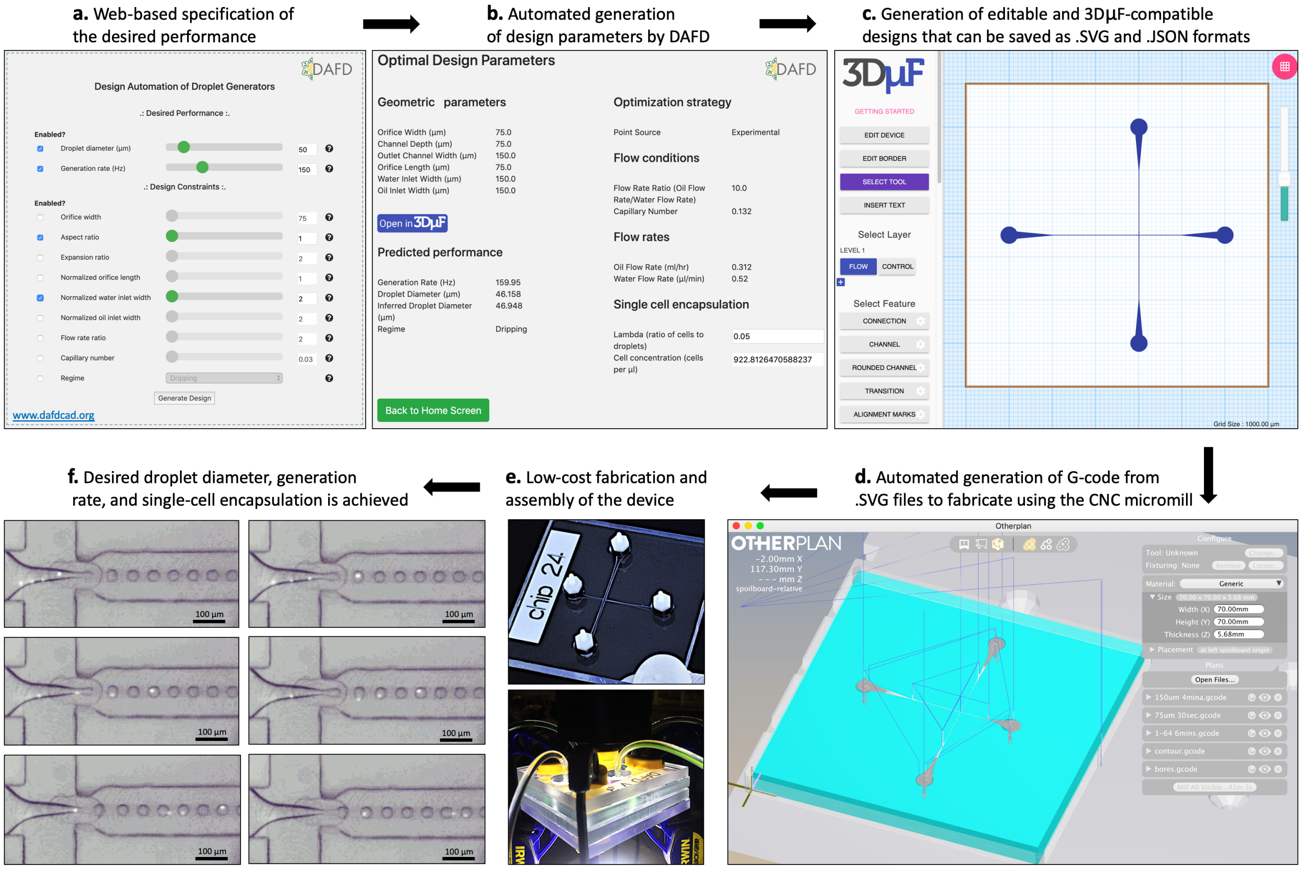


Supplementary Figure 13: The end-to-end workflow of the proposed design automation tool. This end-to-end workflow allows for automated generation of micromill-ready and editable designs that deliver a user-specified performance. **a.** Using a web-based interface the user can specify the desired performance and design constraints. **b.** DAFD provides the required geometry and flow rates that deliver the specified performance. **c.** The generated design can be opened and edited in 3D$\mu$F [10] to generate an SVG file, **d.** which is loaded into the software controlling the micromill to generate G-code for fabrication. **e.** The fabricated device can be assembled in an inexpensive manner [5]. **f.** The flow rates are set to the values proposed by DAFD to achieve the desired performance.

# 2 Supplementary Tables:

Supplementary Table 1: Range of parameters included in the design automation tool. A wide range of design parameters (geometric parameters and flow conditions) are included in this study to generate a standardized and diverse large-scale data-set of design parameters and the associated observed performance (droplet diameter and generation rate).

| **Parameter** | | **Range** | |
|---|---|---|---|
| Name | Unit | Lower bound | Upper bound |
| **Geometry** | | | |
| Orifice width | $\mu$m | 75 | 175 |
| Normalized orifice length | N/A | 1 | 3 |
| Normalized water inlet width | N/A | 2 | 4 |
| Normalized oil inlet width | N/A | 2 | 4 |
| Expansion ratio | N/A | 2 | 6 |
| Aspect ratio | N/A | 1 | 3 |
| **Flow condition** | | | |
| Flow rate ratio | N/A | 2 | 22 |
| Capillary number | N/A | 0.033 | 1.05 |
| **Bounded performance** | | | |
| Droplet diameter | $\mu$m | 25 | 250 |
| Generation rate | Hz | 5 | 500 |

Supplementary Table 2: 25 orthogonal devices proposed by Taguchi design of experiments method are given below ($L_{25}$ Taguchi design). These devices are all tested at the same 34 unique flow conditions to create 850 data-points. During the design automation verification process 18 additional devices were tested to add 148 data-points, totaling 998 data-points in the data-set.

| **Device** | **Geometric parameters** | | | | | |
|---|---|---|---|---|---|---|
| No. | Orifice width ($\mu$m) | Aspect ratio | Expansion ratio | Normalized water inlet width | Normalized oil inlet width | Normalized orifice length |
| #1 | 75 | 1 | 2 | 2 | 2 | 1 |
| #2 | 75 | 1.5 | 3 | 2.5 | 2.5 | 1.5 |
| #3 | 75 | 2 | 4 | 3 | 3 | 2 |
| #4 | 75 | 2.5 | 5 | 3.5 | 3.5 | 2.5 |
| #5 | 75 | 3 | 6 | 4 | 4 | 3 |
| #6 | 100 | 1 | 3 | 3.5 | 4 | 2 |
| #7 | 100 | 1.5 | 4 | 4 | 2 | 2.5 |
| #8 | 100 | 2 | 5 | 2 | 2.5 | 3 |
| #9 | 100 | 2.5 | 6 | 2.5 | 3 | 1 |
| #10 | 100 | 3 | 2 | 3 | 3.5 | 1.5 |
| #11 | 125 | 1 | 4 | 2.5 | 3.5 | 3 |
| #12 | 125 | 1.5 | 5 | 3 | 4 | 1 |
| #13 | 125 | 2 | 6 | 3.5 | 2 | 1.5 |
| #14 | 125 | 2.5 | 2 | 4 | 2.5 | 2 |
| #15 | 125 | 3 | 3 | 2 | 3 | 2.5 |
| #16 | 150 | 1 | 5 | 4 | 3 | 1.5 |
| #17 | 150 | 1.5 | 6 | 2 | 3.5 | 2 |
| #18 | 150 | 2 | 2 | 2.5 | 4 | 2.5 |
| #19 | 150 | 2.5 | 3 | 3 | 2 | 3 |
| #20 | 150 | 3 | 4 | 3.5 | 2.5 | 1 |
| #21 | 175 | 1 | 6 | 3 | 2.5 | 2.5 |
| #22 | 175 | 1.5 | 2 | 3.5 | 3 | 3 |
| #23 | 175 | 2 | 3 | 4 | 3.5 | 1 |
| #24 | 175 | 2.5 | 4 | 2 | 4 | 1.5 |
| #25 | 175 | 3 | 5 | 2.5 | 2 | 2 |

Supplementary Table 3: Performance metrics of scaling law # 1 for predicting the data-set generated in this study. $^*R^2$ Coefficient of determination. ** Root mean squared error. *** Mean absolute percentage error. ****Mean absolute error.

| Parameter | Regime | $R^{2*}$ | RMSE** | MAPE*** | MAE**** |
|---|---|---|---|---|---|
| Droplet diameter | Dripping | 0.446 | 31.7 $\mu$m | 24.5% | 23.3 $\mu$m |
| Droplet diameter | Jetting | -0.6758 | 58.1 $\mu$m | 36.3 % | 46.6 $\mu$m |
| Generation rate | Dripping | 0.350 | 78.7 Hz | 55.2% | 45.8 Hz |
| Generation rate | Jetting | -0.252 | 117.9 Hz | 61.7 % | 80.5 Hz |

Supplementary Table 4: Performance metrics of scaling law # 2 for predicting the data-set generated in this study. *$R^2$ Coefficient of determination. ** Root mean squared error. *** Mean absolute percentage error. ****Mean absolute error.

| Parameter | Regime | $R^{2*}$ | RMSE** | MAPE*** | MAE**** |
|---|---|---|---|---|---|
| Droplet diameter | Dripping | -1.620 | 69.1 $\mu$m | 62.3 % | 59.3$\mu$m |
| Droplet diameter | Jetting | 0.372 | 35.6 $\mu$m | 22.1% | 28.4 $\mu$m |
| Generation rate | Dripping | N.A. | N.A. | N.A. | N.A. |
| Generation rate | Jetting | N.A. | N.A. | N.A. | N.A. |

Supplementary Table 5: Comparison of DAFD predicted and experimentally observed performance for unseen design parameters. These experiments were carried out using a flow-focusing geometry with an orifice width of 75 $\mu$m, aspect ratio of 2.02, expansion ratio of 4.02, normalized orifice width of 2.06, normalized water inlet width of 3.04, and a normalized oil inlet width of 2.94.

| Experiment number | **Flow condition** | | **Predicted** | | **Observed** | |
|---|---|---|---|---|---|---|
| | Capillary number | Flow rate ratio | Droplet diameter ($\mu$m) | Generation rate (Hz) | Droplet diameter ($\mu$m) | Generation rate (Hz) |
| 1 | 0.1 | 3 | 105.1 | 79.7 | 98.1 | 78 |
| 2 | 0.2 | 10 | 49.7 | 303.5 | 55.4 | 260 |
| 3 | 0.15 | 17 | 45.9 | 194.7 | 42.7 | 250 |
| 4 | 0.6 | 5 | 119.3 | 124.1 | 122 | 146 |
| 5 | 0.4 | 9 | 103.3 | 86.7 | 93 | 122 |
| 6 | 0.8 | 22 | 75.2 | 168.8 | 71.7 | 240 |

Supplementary Table 6: DAFD inputs and outputs for droplet diameter specification used to generate Fig. 6.a. of the main manuscript.

| **DAFD input** | | | **DAFD proposed designs** | | | | | | | |
|---|---|---|---|---|---|---|---|---|---|---|
| **Performance** | | **Constraints** | **Geometry ($\mu$m)** | | | | | | **Flow condition** | |
| Diameter | Rate | | Orifice | channel depth | Outlet width | Orifice length | Water inlet | Oil inlet | capillary number | flow rate ratio |
| 25 $\mu$m | None | None | 75 | 75 | 164 | 75 | 151 | 161 | 0.178 | 22 |
| 50 $\mu$m | None | None | 75 | 225 | 450 | 225 | 300 | 300 | 0.132 | 22 |
| 75 $\mu$m | None | None | 75 | 75 | 150 | 75 | 150 | 150 | 0.066 | 6 |
| 100 $\mu$m | None | None | 75 | 150 | 300 | 150 | 225 | 225 | 1.058 | 10 |
| 125 $\mu$m | None | None | 125 | 375 | 375 | 312.5 | 250 | 375 | 0.264 | 16 |
| 150 $\mu$m | None | None | 150 | 375 | 450 | 450 | 450 | 300 | 1.058 | 16 |
| 175 $\mu$m | None | None | 175 | 439 | 706 | 252 | 350.5 | 689.5 | 0.2 | 20 |
| 200 $\mu$m | None | None | 150 | 450 | 600 | 150 | 525 | 375 | 1.058 | 13 |

Supplementary Table 7: DAFD inputs and outputs for droplet diameter and generation rate specification used to generate Fig. 6.b-d. of the main manuscript.

| **DAFD input** | | | **DAFD proposed designs** | | | | | | | |
|---|---|---|---|---|---|---|---|---|---|---|
| **Performance** | | **Constraints** | **Geometry ($\mu$m)** | | | | | | **Flow condition** | |
| Diameter | Rate | | Orifice | channel depth | Outlet width | Orifice length | Water inlet | Oil inlet | capillary number | flow rate ratio |
| **Droplet diameter of 100 $\mu$m** | | | | | | | | | | |
| 100 $\mu$m | 50 | None | 75 | 225 | 450 | 225 | 300 | 300 | 0.264 | 16 |
| 100 $\mu$m | 100 | None | 150 | 300 | 300 | 375 | 375 | 600 | 0.132 | 19 |
| 100 $\mu$m | 150 | None | 100 | 300 | 200 | 150 | 300 | 350 | 0.264 | 13 |
| 100 $\mu$m | 200 | None | 150 | 300 | 300 | 375 | 375 | 600 | 0.264 | 16 |
| 100 $\mu$m | 300 | Orifice=100$\mu$m | 100 | 302.1 | 211.3 | 150 | 300 | 350 | 0.529 | 12.732 |
| 100 $\mu$m | 400 | None | 175 | 262.5 | 350 | 525 | 612.5 | 525 | 1.058 | 22 |
| 100 $\mu$m | 500 | None | 150 | 300 | 300 | 375 | 375 | 600 | 0.529 | 16 |
| **Droplet diameter of 75 $\mu$m** | | | | | | | | | | |
| 75 $\mu$m | 50 | None | 100 | 300 | 200 | 150 | 300 | 350 | 0.066 | 19 |
| 75 $\mu$m | 100 | None | 100 | 300 | 200 | 150 | 300 | 350 | 0.132 | 19 |
| 75 $\mu$m | 150 | None | 100 | 281 | 200 | 150.3 | 299 | 357.8 | 0.2 | 20 |
| 75 $\mu$m | 200 | None | 150 | 150 | 750 | 225 | 600 | 450 | 0.264 | 10 |
| 75 $\mu$m | 300 | None | 100 | 292.9 | 216.9 | 150 | 300.7 | 350 | 0.526 | 22.643 |
| 75 $\mu$m | 400 | None | 100 | 100 | 300 | 200 | 350 | 400 | 1.058 | 16 |
| 75 $\mu$m | 500 | Orifice=100$\mu$m | 100 | 185 | 283 | 197.1 | 350 | 400 | 1.116 | 21.893 |
| **Droplet diameter of 50 $\mu$m** | | | | | | | | | | |
| 50 $\mu$m | 50 | None | 100 | 100 | 300 | 200 | 350 | 400 | 0.066 | 22 |
| 50 $\mu$m | 100 | None | 75 | 112.5 | 225 | 112.5 | 187.5 | 187.5 | 0.132 | 19 |
| 50 $\mu$m | 150 | None | 75 | 225 | 450 | 225 | 300 | 300 | 0.132 | 22 |
| 50 $\mu$m | 200 | None | 75 | 151.5 | 302 | 154.5 | 228 | 220.5 | 0.15 | 14 |
| 50 $\mu$m | 300 | None | 100 | 195.5 | 486.8 | 297.5 | 200 | 244 | 0.15 | 22 |
| 50 $\mu$m | 400 | None | 125 | 125 | 500 | 375 | 312.5 | 437.5 | 0.264 | 22 |
| 50 $\mu$m | 500 | None | 125 | 125 | 500 | 375 | 312.5 | 437.5 | 0.2 | 14 |

# 3 Supplementary Notes:

## 3.1 Supplementary Note 1

A low-cost rapid prototyping technique (i.e., low-cost desktop micromilling) we previously described is used to fabricate all the microfluidic devices [6]. The microfluidic flow-focusing devices were milled out of a 7cm by 7cm polycarbonate substrate with a thickness of 5.56 mm. All the inlet ports were placed 24 mm upstream of the orifice. The inlet width are set to the designed width starting from 10 mm upstream of the orifice, to allow the flow to be fully-developed. The inlet channel widths decrease from the port diameter to design width gradually as shown in Supplementary Fig. 1. The outlet width was kept at the designed outlet width up to 5 mm downstream of the orifice and then was gradually increased to match the outlet port diameter. The original design file was created in SolidWorks and is defined in a way that the users would only need to update the values for the six geometric parameters without having to edit or redraw any additional geometries. The original and editable design file is available on Metafluidics and can be also opened and edited in 3D$\mu$F. The designs are saved as .STEP files and are loaded to Fusion 360 to generate G-codes necessary to fabricate the microfluidic device using the low-cost CNC mill (this can also be achieved by using 3D$\mu$F software and downloading an SVG, and loading it directly to the CNC mill to automatically create G-code without using Fusion 360, although less control over the G-code is a drawback). The G-codes are then loaded on the CNC machine to mill out micro-channels of the flow layer. DAFD is also integrated with 3D$\mu$F to allow for the DAFD suggested designs to be opened up in 3D$\mu$F environment. Using 3D$\mu$F researchers can edit the designs or connect the droplet generator to available microfluidic components and finally download the files as SVG files. The SVG files can be directly loaded to the software controlling the CNC mill (Otherplan/BantamTools) that automatically generates the GCODE necessary to fabricate the microfluidic device. The control layer does not include any milled features since our design is valve-less. A thin (250-500 $\mu$m) layer of PDMS (Slygard 184) is sandwiched between the flow and control layer to seal the device. In order to improve the sealing pressure two layers pressure distributors are milled out of polycarbonate (with holes to allow for ports and tubes to be connected) and clamped down to deliver a uniform seal and finish the assembly process. Using this method each device costs less than $10 and can be fabricated in less than one hour.

## 3.2 Supplementary Note 2

The six parameters defining a microfluidic flow-focusing droplet generator, were varied according the values observed in the literature while considering the fabrication limits of low-cost micromilling (75 $\mu$m minimum feature size). All the geometric parameters were normalized to the orifice width (except the orifice width itself). The range of the parameters used in this study to create the data-set are given in Supplementary Table 1. A total 43

unique flow-focusing geometries (i.e, 25 orthogonal geometries as previously described [5] and given in Supplementary Table 2, with 18 additional geometries added until accurate design automation was achieved) were fabricated for this study. The 25 orthogonal devices were tested at the same 34 unique flow conditions, creating 850 data-points. The remaining 18 devices and 148 data-points were added during verification process of the design automation tool.

Droplet break-up occurs when the viscous forces exerted by the continuous phase (oil) overcome the surface tension of the dispersed phase (water) [1]. Therefore, capillary number, which represents the ratio of viscous forces to surface tension, is a suitable dimensionless number to characterize droplet formation [12]. Capillary number captures geometry, fluid properties, and the flow rate of the continuous phase [5], and can be approximated by Supplementary Equation (1):

$$Ca. = \frac{\mu_c W_d Q_c}{\sigma H \cdot W_c} \left[ \frac{1}{Or} - \frac{1}{2 \cdot W_c} \right], \tag{1}$$

where $\mu_c$ represents dynamic viscosity, $Q_c$ is oil flow rate, $\sigma$ denotes surface tension between the continuous and dispersed phases, $H$ is channel depth, and $W_d$, $W_c$, and $Or$ are water inlet, oil inlet, and orifice widths, respectively. In flow-focusing droplet formation, capillary number typically varies between 0.001 to 1 [4, 14]. For a given flow-focusing geometry, capillary number, and fluid properties, the flow rate of the continuous phase can be calculated using Supplementary Equation (1). The flow rate of the dispersed phase can be determined using the flow rate of the continuous phase and the flow rate ratio. Capillary number and flow rate ratio were varied according to the values observed in the literature and were used to calculate the oil and water flow rates for the designed flow-focusing geometries.

Each device is also tested over a wide range of flow conditions (i.e., combination of capillary number and flow rate ratio). Capillary number and flow rate ratio are the two main dimensionless numbers used to characterize the performance of microfluidic droplet generation. Therefore, these two dimensionless numbers were varied according the range observed in the literature. Capillary number was varied to also ensure comparable number of data-points are recorded in both the dripping regime and jetting regime. Flow rate ratio was varied and relatively high flow rate ratio were also included to achieve smaller droplet diameters and compensate for the fact that orifice widths smaller than 75 $\mu$m could not be reliably fabricated. The large flow rate ratios, allowed for droplets with diameters about one-third of the orifice width to be generated. The range of the capillary number and flow rate ratios used in this study to create the data-set is given in Supplementary Table 1. Each device was tested at a maximum number of 34 unique flow conditions and minimum of 1. A total 65 unique flow conditions were included in our data-set as shown in Supplementary Fig. 2

### 3.3 Supplementary Note 3

Python Scikit-learn and Keras packages were used to implement and analyze the neural network models [2]. For the regime classification, the data-set was normalized and split to train and test sets (80%−20% ratio) to later evaluate the accuracy of performance prediction on the test-set. To avoid over fitting to the train-set, which causes a poor prediction for the unseen data-points on the test-set, a 10-fold cross validation approach is used. In summary, we partition the train-set (80% of the data-points) into 10 subsets (i.e., folds) and iteratively train the model on 9 folds while using the remaining fold (i.e., 1 holdout fold) as the validation-set. The developed neural network model had the following structure: input layer with 8 neurons (8 inputs as the geometry and flow condition features), 3 hidden layers with 32-16-16 neurons having Rectified Linear Unit (ReLU) activation functions, and the output layer with 1 neuron (1 output that determines the predicted regime) having Sigmoid activation function. The binary cross entropy loss function was minimized using Keras built-in Root Mean Square Propagation (RMSProp) optimizer:

$$\text{Cross Entropy} = -\frac{1}{n}\sum_{i=1}^{n} y_i log(\hat{y}_i) + (1 - y_i)log(1 - \hat{y}_i), \tag{2}$$

where $y_i$ is the observed class corresponding to the $i^{th}$ data in the input and $\hat{y}_i$ is the corresponding predicted class and $n$ is the total number of data-points on the train-set. Figure 3 demonstrates the decrease in the binary cross entropy loss function for the classification problem over train and test-sets.

Droplet diameter and generation rate prediction is formulated as a regression problem, the same normalization and splitting process as in the classification was applied to each regime's data-set. The neural network structure for predicting generation rate in jetting regime and droplet diameter in both regimes, were similar to the structure of the model used for regime prediction with input layer having 8 neurons (8 inputs as the geometry and flow condition features), 3 hidden layers with 32-16-16 neurons having Rectified Linear Unit (ReLU) activation functions, and the output layer with 1 neuron (1 output that determines the droplet diameter or generation rate) having linear activation function. In the case of the neural network for predicting generation rate in dripping regime, another hidden layer was added due to complexity of the regression problem and neurons in hidden layers were as 32-16-16-8 respectively. Mean squared error (MSE) was considered as the loss function and minimized using the adaptive learning rate optimization algorithm called Adam optimizer.

$$\text{MSE} = \frac{1}{n}\sum_{i=1}^{n}(\hat{y}_i - y_i)^2, \tag{3}$$

where $y_i$ is the $i^{th}$ data-point observed generation rate or droplet diameter, and $\hat{y}_i$ is the corresponding predicted value. Adam is a combination of AdaBoost algorithm and RMSProp which uses momentums to further improve the search for the loss function minimizer [9].

Figure 4 demonstrates decrease in the mean squared error loss function for regression models (droplet diameter and generation rate prediction in both dripping and jetting regimes) over train and test sets.

To increase the convergence speed and computational efficiency, optimizers were implemented using batch method with proper batch size. Another approach employed to avoid over fitting of the model to the train-set is Early Stopping [8]. Given a large epoch number for training, we stop further iterations of parameter tuning if further iterations cause a deviation between the performance of the model on the train and validation sets (that means the model is fitting the train-set too well which will prevent it to generalize to the test-test data). More information on classification and regression with neural networks can be found in [3],[11].

## 3.4 Supplementary Note 4

Scaling laws are great and simple tools for understanding the overall effect of major parameters on the performance of microfluidic droplet generators. Scaling laws were not initially proposed for accurate performance prediction, however, due to a lack of an alternative their performance prediction accuracy was compared to DAFD's accuracy.

It should be mentioned that although the flow-focusing geometries used in scaling laws #1 and #2 is identical at the point of droplet generation, the oil inlet orientation up-stream of the orifice is different. Nonetheless, by using entrance length approximation it can be demonstrated that the effect of the oil inlet orientation up-stream of the orifice can be neglected. In both studies, the oil inlets are not exactly placed perpendicular to the water inlet channels, the oil flow channel itself becomes perpendicular to the water flow channel upstream of droplet generation (right before the orifice). Therefore, as long as the flow inside the perpendicular section of the oil inlet channel is fully-developed it can be assumed that the shape of the inlet upstream of the perpendicular section does not affect droplet generation. It is the assumption of a fully-developed flow that allows for any scaling law to be proposed and used, otherwise, the shape of inlets, tubing, syringes, and connectors would invalidate scaling laws. A flow in a channel will be fully-developed after it passes an entrance-length. The entrance length in a laminar flow inside a rectangular channel can be estimated by Supplementary Equation (4):

$$L_{ent.} \approx 0.05\,\mathrm{Re}\,.D_h, \tag{4}$$

where Re is the Reynolds number and $D_h$ is the hydraulic diameter defined by Supplementary Equation (5) in rectangular cross-sections:

$$D_h = \frac{4A}{P} = \frac{2WH}{W+H}, \tag{5}$$

where $W$ is the oil inlet width and $H$ is the channel depth. Since the Reynolds number is typically less than one in microfluidics the flow is deemed to be laminar (for our devices the average Re number was less than 0.5). By assuming a relatively large channel dimension of 700*700 $\mu$m (worst-case scenario, i.e., largest oil inlet channel in our study); the hydraulic diameter of the oil inlet channel will be 700 $\mu$m. Additionally, by even assuming a Reynolds number of Re = 1, the entrance length can be approximated to be less than 35 $\mu$m. Therefore, if the length of the perpendicular section of oil inlet right before the orifice is larger than a couple of tens of micrometers it is safe to assume the shape of the channels upstream of the perpendicular section of the oil inlet has a minimal effect on droplet generation. It should be noted that the channel widths and depth used in the studies of references [7] and [14], are much smaller than our assumed worst-case scenario, therefore, the entrance length is expected to be only a couple of micrometers in those studies.

The perpendicular section of the oil inlet in the flow-focusing geometry used in scaling laws #1 and #2 is larger than a couple of tens of microns. Additionally, since the dimensions used in their study is much smaller than our assumed worst-case scenario, the entrance length is expected to be much smaller than 35 $\mu$m. Therefore, it is safe to assume that the orientation of the channels upstream of the perpendicular section of the oil inlet has a minimal effect on the process of droplet generation.

### Scaling law # 1

This scaling is proposed by Lee et al [7]. In the mentioned study scaling laws for both droplet diameter and generation rate of flow-focusing devices were proposed. It was shown that the normalized droplet diameter (normalized to the hydraulic diameter) scales with capillary number. The hydraulic diameter was defined by Supplementary Equation (6), and the scaling law was proposed for droplet diameter as given in Supplementary Equation (7):

$$D_{\mathrm{h}} = \frac{2W_{\mathrm{or.}} \cdot h}{W_{\mathrm{or.}} + h}, \tag{6}$$

where $D_{\mathrm{h}}$ is the hydraulic diameter, $W_{or.}$ is the orifice width, and $h$ is the channel depth.

$$\frac{D}{D_{\mathrm{h}}} = C \cdot (Ca.)^{-0.47} \tag{7}$$

This equation is proposed for all droplet formation regimes for over a wide range of viscosity ratios (from 0.0028 to 0.048). The viscosity ratio used in our study (i.e., mineral oil and DI water) was 0.017, thus, within the range of Lee et al. study. One, challenge in using scaling laws for performance prediction is generating a data-set to find a proper fitting constant (i.e., $C$ in Supplementary Equation (7)). Therefore, using scaling laws for performance prediction requires a data-set to find the constants used in the proposed equations. We found different constants to be better fitted to our data-set for different regimes (dripping and jetting regimes). For predicting the droplet diameter in the dripping regime using Supplementary Equation (7), we found a constant of $C_{\mathrm{Dripping}} = 0.18$ to better fit our data. However, for

predicting the droplet diameter in the jetting regime using Supplementary Equation (7), we found a constant of $C_{\text{Jetting}} = 0.4$ to better fit our data. The plots generated in Fig. 3.a. of the main manuscript were generated using these constants.

Additionally, Lee et al. proposed a scaling law for predicting the generation rate of flow-focusing droplet generators. It was shown that the normalized time (normalized to the dispersed phase flow rate and the hydraulic diameter to the power of three) required to form a droplet scales with capillary number as given in Supplementary Equation (8).

$$\frac{\tau \cdot Q_{\text{w}}}{{D_{\text{h}}}^3} = C.(Ca.)^{-1} \tag{8}$$

Therefore, by calculating the time required to form a droplet ,$\tau$, from the scaling law, the generation rate can be calculated using Supplementary Equation (9):

$$F = \frac{1}{\tau}, \tag{9}$$

where F is the generation rate in droplets per second (Hz). Similar to predicting the droplet diameter using scaling law # 1, we found different constants to better fit the generation rate of dripping and jetting regimes. For predicting the generation rate in the dripping regime using Supplementary Equation (9), we found a constant of $C_{\text{Dripping}} = 0.012$ to better fit our data. However, for predicting the generation rate in the jetting regime using Supplementary Equation (9), we found a constant of $C_{\text{Jetting}} = 0.2$ to better fit our data. The plots generated in Fig. 3.a. of the main manuscript were generated using these constants. The performance metrics for scaling # 1 is given in Supplementary Table 3.

### Scaling law # 2

This scaling law is proposed by Ward et al [14]. In the mentioned study a scaling for predicting the droplet diameter of a flow-driven flow-focusing droplet generator, while using mineral oil and DI water, is proposed. It was shown that the normalized droplet diameter (normalized to the width of the orifice) scales with the flow rate ratio as given in Supplementary Equation (10):

$$\frac{D}{W_{\text{or.}}} = 2.35\left(\frac{Q_{\text{w}}}{Q_{\text{o}}}\right)^{0.25}, \tag{10}$$

where $D$ is the droplet diameter, $W_{\text{or.}}$ is the orifice width, $Q_{\text{w}}$ is water flow rate, and $Q_{\text{o}}$ is the oil flow rate.

Additionally, Ward et al. proposed a scaling law for generation rate based on the droplet velocity and the distance between two consecutive droplets as given in Supplementary Equation (11):

$$F = \frac{1.15 \cdot U}{d}, \tag{11}$$

where $F$ is generation rate, $U$ is droplet velocity, and $d$ is the distance between two consecutive droplets. Since this equation is based on the observed droplet velocity and the distance between two consecutive droplets, it cannot be used for performance prediction. Therefore, for plotting Fig. 3.a. of the main manuscript, only Scaling law #1 is used for predicting the generation rate. The performance metrics for scaling # 2 is given in Supplementary Table 4.

Apart from the fluid dynamics, the scaling laws are typically plotted in a log-log scale, which is well in-line with their indented purpose, which is demonstrating the overall effect of major parameters on the output of interest. As shown in Supplementary Fig. 5, when plotting the same data as in Fig. 3 of the main manuscript on a log-log scale some interesting conclusions can be drawn. First, it can be concluded as mentioned in the manuscript the discussed scaling laws are great in capturing the overall effect of parameters on performance. This can be clearly seen in Supplementary Fig. 5 where both scaling laws successfully captured the overall trend of the DAFD dataset on a log-log scale. Even, when comparing to the data published in the original research articles, it can be concluded that the scaling laws show approximately similar accuracies in capturing the overall change in performance, whether using their own dataset or the dataset generated in this study.

## 3.5 Supplementary Note 5

The unseen design parameter experiments were carried on a geometry that only contributed one data-point with a flow condition of Ca. number of 0.047 and flow rate ratio of 19.37 to the data-set. This geometry had an orifice width of 75 $\mu$m, aspect ratio of 2.02, expansion ratio of 4.02, normalized orifice width of 2.06, normalized water inlet width of 3.04, and a normalized oil inlet width of 2.94. To assess the accuracy of DAFD for predicting the performance while using unseen design parameters, six new experiments with unseen flow conditions were carried out and the values predicted by DAFD were then compared to the experimentally observed performance with the same flow conditions. The flow conditions of each experiment and the results of this comparison is given in Supplementary Table 5.

## 3.6 Supplementary Note 6

A significant barrier to entry for machine learning adoption in many computational workflows is that it requires domain specific knowledge, as well as expertise on the particular machine learning models used to enable accurate performance prediction.

Tuning a neural network can be tedious and time consuming. On the other hand, tuning the hyper-parameter is analogous to an optimization process that in the most naive way can be solved by iteratively searching through the entire parameter space. While doing this can ensure the most optimal result, it is in reality difficult to do, as the computational cost grows exponentially as new parameters are introduced. Alternatively, one can employ a method that evaluates some number of random combinations in the parameter space, and

iteratively pick a new best-value until stopping criteria (e.g. number of iteration, or parameter combination) is met. The former approach is referred to as grid search, while the latter is referred to as random search. Neural Optimizer supports two hyper-parameter tuning approaches, namely grid search and random search. While the most optimum combination of parameters is guaranteed through grid Search, it suffers from the curse of dimensionality, which makes it impractical for neural networks with high dimensions. On the other hand, random search offers more efficient approach for hyper-parameter optimization than trials on a grid, with comparable performance to grid search but within a small fraction of computation time. Thus, for a larger networks, hyper-parameter tuning based on random search is recommended.

DAFD Neural Optimizer enables researchers to upload a data-set and specify the target variable and design parameters for both regression and classification models (see Supplementary Fig. 6). Once, the parameters are specified Neural Optimizer allows for an automated data-to-model machine learning framework. Neural Optimizer supports both Grid and Random search for hyper-parameter, and network architecture tuning. The tunable parameters include the number of hidden layers, the number of nodes for each hidden layer, the number of epochs, and the batch size (see Fig. 7). In this work, we built five different neural networks, consisting of one binary classifier for regime classification, and four different regressors for predicting the droplet generation rate and size for each of the regime classes. Initially, we designed and tuned these networks in an ad-hoc manner, to get the best configurations that yield the most optimal performances in several trials (in terms of accuracy, and F-score for the classifier and MAE, RMSE, and r-squared for the regressors). Consecutively, to evaluate the performance of DAFD Neural Optimizer we employed it for automated generation of neural networks, by specifying the predictors and target variable, type of models (classification or regression), and the hyper-parameter space. Even though we obtained different configurations, we empirically demonstrated that the software-optimized networks can perform similarly to the ones manually tuned, as shown in Fig. 5a of the main manuscript.

## 3.7 Supplementary Note 7

Transfer learning generally exploits an already trained task to transfer knowledge and improve the generalization in a related task. Transfer learning [13] is a relatively new area in machine learning and deep learning, allowing researchers to train accurate models on a large data-set or use the already trained and accurate models, known as pre-trained models, as a basis for developing a new model in a similar data-set. The idea comes from the fact that the earlier layers of a neural network contain more generic features and the later layers become progressively more specific to the details of the the data-set used in the training stage. In our application, we can justify that if a model can predict the regimes based on the droplet features given that the droplet is water, it can also predict the regimes when droplets are oil ( if the features of droplets do not change drastically). Therefore, for a given data-set by

the user, we can keep the earlier layers from the pre-trained models on our large data-set to extract the generic features of the new data-set. If the new data-set is small or different from our original data-set, we can choose to fine tune or re-train the last few layers of the the pre-trained models (updating) to capture the specific features of the new data-set and avoid over-fitting.

To do so, the structure and weights of the layers in the optimized pre-trained models are saved using Keras package. For the new data-set provided by the user, we freeze the first 2 layers of the pre-trained model by disabling the trainability of the weights. The remaining layers are set to be trainable to fit the new data-set.

## 3.8 Supplementary Note 8

We combined predictive and generative models trained on our experimental data to provide researchers with an easy-to-use design automation tool for microfluidic droplet generators. This tool, can suggest the device geometry and flow condition that delivers the desired performance, thus, eliminating the need for costly and time-consuming design iterations.

In addition to satisfying desired performance requirements, DAFD can also take in optional design constraints. These constraints can be added to clamp the suggested design parameters to a specified range or a specific value. For example, if a constraint of 0.5 - 0.6 is applied to the capillary number, the returned designed device will perform at a capillary number in that range. If an orifice width of 150 $\mu$m is specified, the returned design parameters will be forced to have that orifice width exactly. DAFD also supports multiple user-specified constraints, which allows researchers to specify their desired performance while DAFD proposed design is specifically tailored towards the needs of the user.

The design automation procedure can be divided into two main parts: A) finding an experimental point with a performance most closest the desired performance and design constraints (if specified) and B) adjusting and optimizing the design parameters of that experimental point to achieve the desired performance.

Finding an initial experimental point allows DAFD to start in an area of the design space which is known to be close to the user specified desired performance. Every point in the bounded experimental data-set is considered (888 points in total). Points which do not meet the following criteria Supplementary Equation (12) are immediately discarded:

$$\begin{aligned} &i.\ \text{Experimental regime} = \text{Predicted regime} \\ &ii.\ \text{Experimental regime} = \text{Constrained regime} \\ &iii.\ \text{Constrained orifice} < \text{Desired diameter} \implies \text{Experimental regime} = \text{Jetting}, \end{aligned} \tag{12}$$

where "Experimental" refers to the actual observed value in the data-set. "Predicted" refers

to the neural network models predictions given the flow conditions and geometries of the experimental point. "Constrained" refers to the optional user-specified design constraints placed on a parameter. If there are no constraints on the regime or the orifice size, the second and third criteria are ignored. The first criteria is added to ensure the neural network models are accurately predicting the droplet formation regime of that data-point (therefore, both curating the data-set and predictive models to avoid the data-points where the model and data-set are not compatible) The second criteria is added to ensure that only the data-points with the user-specified desired regime are considered when the user has specified a certain droplet formation regime (dripping or jetting). The third criteria is added for the cases that the user has specified both a droplet diameter and an orifice width and the droplet diameter is larger than the orifice width. This criteria ensures that the optimization algorithms will look only into data-points with jetting regime (where the droplet diameter is typically larger than the orifice width) to increase the accuracy and efficiency of the algorithms.

Points that meet the above criteria are then ranked according to our first cost function Supplementary Equation (13):

$$\begin{aligned} C(X) = |D_{\text{desired}} - D_{\text{predicted}}| + |F_{\text{desired}} - F_{\text{predicted}}| \\ + \sum_{T \in U} 1000 + |X_T - T_{\text{avg}}| \end{aligned} \tag{13}$$

where C(X) is the cost function used to rank the data-points, D is the droplet diameter, F is the generation rate, U is the *unmet* constraints (constraints which the experimental point does not satisfy), $X_T$ is the value of the constrained parameter for a given data-point, and $T_{\text{avg}}$ is the middle point of the constraint range for a parameter. 1000 is simply a very large number which will ensure that points which actually meet the specified constraints are considered first. All of the constraints and design parameters are transformed to fit a normal distribution. This ensures that parameters with generally higher values (such as orifice width) do not contribute more to the cost function than parameters with lower values (such as capillary number).

Using Supplementary Equation(13), the experimental point with the lowest cost is then returned (i.e., raw experimental point return ). If the experimental point with the lowest cost meets an acceptable tolerance from the desired performance, then we return that point to the user without any further modifications. The raw experimental point return will prevent unnecessarily running the DAFD which might introduce errors because of the minor inaccuracies in the predictive models. The tolerances for droplet diameter are set to be lower than the average error of the predictive models (given in Table 1 of the main manuscript), as given by Supplementary Equation (14):

$$
\begin{aligned}
&i.\ X_T \in C_T \forall C_T \\
&ii.\ |D_{\text{desired}} - D_{\text{experimental}}| < 5\mu\text{m} \\
&iii.\ |D_{\text{desired}} - D_{\text{predicted}}| < 10\mu\text{m} \\
&iv.\ |D_{\text{desired}} - D_{\text{inferred}}| < 10\mu\text{m}
\end{aligned}
\tag{14}
$$

The first criteria ensures that all design parameters $X_T$ are within the user-specified constraint range $C_T$ if such a constraint is imposed. The second criteria requires the observed experimental droplet diameter of the data-point to be within 5 $\mu$m of the desired droplet diameter. The third criteria ensures that the predicted droplet diameter for that data-point is within the tolerance of the predictive neural network models (is less than 10 $\mu$m). The fourth criteria requires the droplet diameter inferred from the flow conditions and predicted generation rate to be within 10 $\mu$m of the desired diameter (the tolerance of the predictive models given in Table 1 of the main manuscript). The flow rate (the volumetric flow of fluid per second) can be divided by the generation rate (the number of droplets generated per second) to get the average volume per droplet. By assuming a spherical droplet, we can obtain an inferred average droplet diameter, as given in Supplementary Equation (15):

$$
D_{\text{inf.}} = 10^6 \cdot \sqrt[3]{\frac{Q_{\text{w}}}{F_{\text{pred.}}} \cdot \frac{6}{\pi}}\ , \tag{15}
$$

where $D_{\text{inf.}}$ is the inferred droplet diameter in $\mu$m (assuming a uniform droplet size) and $Q_{\text{w}}$ is the dispersed phase flow rate in $m^3/s$. By adding the forth criteria the algorithm entangles the predictive models for both droplet diameter and generation rate together, to avoid design spaces where either one or both models are less accurate. Thus, allowing the design automation accuracy to surpass the accuracy of the predictive models in most cases.

In addition to the criteria for raw experimental point return from the droplet diameter using Supplementary Equation (14), the deviation in generation rate of the returned data-point and the desired generation rate is also bounded. If the specified desired generation rate is below 100 Hz, the predicted and observed generation rate of the experimental data-point must be within 15 Hz of the desired rate. If the desired generation rate is above 100 Hz, the predicted and the observed generation rate of the experimental data-point must be within 15% of the desired rate. These values (15 Hz and 15%) are less than the tolerances of the predictive models (given in Table 1 of the main manuscript). Therefore, if a data-point exists on the the data-set that has an observed performance close to the user-specified desired performance, that data-point will be returned without needing running an optimization algorithm (which is prone to the minor inaccuracies of the predictive models).

If the experimental point with the minimum evaluated cost does not meet the raw return criteria for droplet diameter and generation rate, the second experimental point with the lowest cost is checked against the raw return criteria, then the third, and so on. If no experi-

mental point meets the raw return criteria, then a DAFD algorithm is initiated to propose a new design (geometry and flow condition) that would deliver the user-specified performance, while conforming to the user-specified constraints. To achieve design automation, DAFD will minimize a new cost function as given in Supplementary Equation (16):

$$\begin{aligned} C(X) = |D_{\text{desired}} - D_{\text{predicted}}| + |D_{\text{desired}} - D_{\text{inferred}}| + \\ + |F_{\text{desired}} - F_{\text{predicted}}| \end{aligned} \tag{16}$$

To generate a new design to achieve the user-specified performance, DAFD takes the initial design parameters from data-point closest to the desired performance and adds and subtracts some small normalized step-size $\epsilon$ (which we set to 0.001) to each of the normalized design parameters. This results in 16 slightly different designs (8 total parameters with 6 geometric and 2 flow condition parameters with $\epsilon$ added and subtracted to them, thus 16 new designs). The modified design that reduced the cost function the most is taken as the new initial design for the next iteration. This process is then repeated until the cost function reaches zero, or for 5000 steps or until the modifications fail to decrease the cost function by more than 0.0001 (whichever comes first). The final design iteration is then returned to the user. This workflow is further demonstrated in Supplementary Fig. (9).

Additionally, if the user had specified design constraints, that parameter would be fixed and not adjusted during the design automation process. For example if the user specifies an orifice width of 125 $\mu$m, then during the design automation process 14 new designs will be generated during each iteration (7 parameters in total with $\epsilon$ added and subtracted to them, thus 14 new designs). An example work-flow of when the user specifies a constraint on the orifice width is illustrated in Supplementary Fig. 10.

## 3.9 Supplementary Note 9

DAFD allows the users to specify the desired performance and design constraints in an easy to use interface, as shown in Supplementary Fig.(11. Once, the desired performance and design constraints are specified, DAFD provides the geometry, flow condition, and cell concentration (if single-cell is specified) in return as shown in Supplementary Fig. 12. In this section, the droplet generator designs proposed by DAFD design automation are introduced for specifying only droplet diameter or both droplet diameter and generation rate.

#### Diameter specification

The inputs of DAFD (specified performance) and the outputs of DAFD (geometry and flow condition) for droplet droplet specification used to generate Fig. 6.a. of the main manuscript and verify DAFD are given in Supplementary Table (6).

**Diameter and generation rate specification**

The inputs of DAFD (specified performance  constraints) and the outputs of DAFD (geometry and flow condition) for droplet droplet and generation rate specification used to generate Fig. 6.b-d. of the main manuscript and verify DAFD are given in Supplementary Table (7). For some cases the orifice width is constrained to a specific value (the cutting diameter of the available endmills: 75, 100, 125, 150, 175 $\mu$m) to ensure a high fabrication accuracy [6]. The value is determined by running DAFD once without the constraint, and then running DAFD again while constraining the orifice width to the endmill cutting diameters (both smaller and larger) closest to the previously DAFD proposed orifice width. The design that resulted in an inferred diameter closest to the specified diameter was picked, fabricated, and tested.

## 3.10 Supplementary Note 10

DAFD enables a smooth transition from high-level specification to a working prototype, as shown in Fig. 13. Once a microfluidic flow-focusing design is suggested by DAFD based on the user-specified performance, researchers can use the DAFD-3D$\mu$F module on the website (open in 3D$\mu$F button). This would import the suggested design to 3D$\mu$F which an online interactive microfluidic CAD tool [10]. The users can download their design in a format that is compatible with micromilling (.SVG files). These files can be loaded to the software controlling the micromill (e.g. Bantam Tools) to automatically create Gcodes or load the files to CAM software (e.g. Fusion360) for a more advanced control on the Gcodes. Once Gcodes are created the device can be fabricated in a mostly automated manner [6, 5] using a low-cost desktop micromill and assembled as explain is Supplementary Note 1.

# Supplementary References


[1] Charles N Baroud, Francois Gallaire, and Rémi Dangla. Dynamics of microfluidic droplets. *Lab on a Chip*, 10(16):2032–2045, 2010.

[2] François Chollet et al. Keras. https://keras.io, 2015.

[3] Stephan Dreiseitl and Lucila Ohno-Machado. Logistic regression and artificial neural network classification models: a methodology review. *J. Biomed. Inform.*, 35(5-6):352–359, 2002.

[4] Piotr Garstecki, Howard A Stone, and George M Whitesides. Mechanism for flow-rate controlled breakup in confined geometries: A route to monodisperse emulsions. *Physical review letters*, 94(16):164501, 2005.

[5] Ali Lashkaripour, Christopher Rodriguez, Luis Ortiz, and Douglas Densmore. Performance tuning of microfluidic flow-focusing droplet generators. *Lab on a Chip*, 19(6):1041–1053, 2019.

[6] Ali Lashkaripour, Ryan Silva, and Douglas Densmore. Desktop micromilled microfluidics. *Microfluidics and Nanofluidics*, 22(3):31, February 2018.

[7] Wingki Lee, Lynn M Walker, and Shelley L Anna. Role of geometry and fluid properties in droplet and thread formation processes in planar flow focusing. *Physics of Fluids*, 21(3):032103, 2009.

[8] Lutz Prechelt. Early stopping-but when? In *Neural Networks: Tricks of the trade*, pages 55–69. Springer, 1998.

[9] Sebastian Ruder. An overview of gradient descent optimization algorithms. *arXiv preprint arXiv:1609.04747*, 2016.

[10] Radhakrishna Sanka, Joshua Lippai, Dinithi Samarasekera, Sarah Nemsick, and Douglas Densmore. 3d $\mu$ f-interactive design environment for continuous flow microfluidic devices. *Scientific reports*, 9(1):1–10, 2019.

[11] Donald F Specht. A general regression neural network. *IEEE transactions on neural networks*, 2(6):568–576, 1991.

[12] Shia-Yen Teh, Robert Lin, Lung-Hsin Hung, and Abraham P Lee. Droplet microfluidics. *Lab on a Chip*, 8(2):198–220, 2008.

[13] Lisa Torrey and Jude Shavlik. Transfer learning. In *Handbook of research on machine learning applications and trends: algorithms, methods, and techniques*, pages 242–264. IGI Global, 2010.

[14] Thomas Ward, Magalie Faivre, Manouk Abkarian, and Howard A Stone. Microfluidic flow focusing: Drop size and scaling in pressure versus flow-rate-driven pumping. *Electrophoresis*, 26(19):3716–3724, 2005.
