# Supplementary material for: Machine learning enables design automation of microfluidic flow-focusing droplet generation
Source: Nat Commun. 2021 Jan 4;12:25. doi: 10.1038/s41467-020-20284-z (PMC7782806; doi:10.1038/s41467-020-20284-z)
Supplement: Supplementary file 3 — Description of Additional Supplementary Files [file 41467_2020_20284_MOESM3_ESM.pdf]

Description of Additional Supplementary Files

File Name: Supplementary Movie 1
Description: DAFD Design Automation Tool Tutorial
